# Effects of tofacitinib therapy on arginine and methionine metabolites in association with vascular pathophysiology in rheumatoid arthritis: A metabolomic approach

**DOI:** 10.3389/fmed.2022.1011734

**Published:** 2022-11-10

**Authors:** Boglárka Soós, Attila Hamar, Anita Pusztai, Monika Czókolyová, Edit Végh, Szilvia Szamosi, Zsófia Pethő, Katalin Gulyás, György Kerekes, Sándor Szántó, Gabriella Szűcs, Uwe Christians, Jelena Klawitter, Tamás Seres, Zoltán Szekanecz

**Affiliations:** ^1^Department of Rheumatology, Faculty of Medicine, University of Debrecen, Debrecen, Hungary; ^2^Intensive Care Unit, Department of Internal Medicine, Faculty of Medicine, University of Debrecen, Debrecen, Hungary; ^3^Department of Sports Medicine, Faculty of Medicine, University of Debrecen, Debrecen, Hungary; ^4^Department of Anesthesiology, University of Colorado Anschutz Medical Campus, Aurora, CO, United States

**Keywords:** rheumatoid arthritis, tofacitinib, atherosclerosis, metabolomics, arginine, methionine

## Abstract

**Introduction:**

Rheumatoid arthritis (RA) has been associated with changes in lipid, arginine and NO metabolism with increased cardiovascular (CV) risk. The aim of this study is to evaluate the effect of tofacitinib, a Janus kinase (JAK) inhibitor, on arginine and methionine metabolism in correlation with inflammation, functional and pathological vascular changes during one-year treatment of patients with RA.

**Materials and methods:**

Thirty RA patients with active disease were treated with either 5 mg bid or 10 mg bid tofacitinib for 12 months. We determined DAS28, CRP, IgM rheumatoid factor (RF) and anti-cyclic citrullinated peptide (CCP) levels. We assessed brachial artery flow-mediated vasodilation (FMD), carotid intima-media thickness (IMT) and pulse-wave velocity (PWV) by ultrasound at baseline and after 6 and 12 months. We also determined plasma L-arginine, L-citrulline, L-ornithine, inducible nitric oxide synthase (iNOS), asymmetric (ADMA) and symmetric dimethylarginine (SDMA), L-N-monomethyl-arginine (L-NMMA), cysteine, homocysteine, and methionine levels at these time points.

**Results:**

Twenty-six patients (13 on each arm) completed the study. CRP, ESR and DAS28 decreased significantly during one-year treatment with tofacitinib. Arginine and ADMA showed a negative univariate correlation with CRP but not with FMD, PWV or IMT. Tofacitinib at 10 mg bid significantly increased L-arginine, L-ornithine, iNOS and methionine levels after 12 months. ADMA and SDMA levels did not change in our study. Methionine showed negative correlation with FMD at baseline and positive correlation with PWV after 12 months. No change was observed in FMD and PWV but a significant increase was measured in IMT at 6 and 12 months. Multivariate analysis indicated variable correlations of L-arginine, L-citrulline, ADMA, L-NMMA, homocysteine and methionine with DAS28, CRP, ESR and RF but not with anti-CCP after one-year treatment. With respect to vascular pathophysiology, only PWV and methionine correlated with each other.

**Conclusion:**

One-year tofacitinib treatment suppressed systemic inflammation and improved functional status in RA. FMD, PWV have not been affected by one-year tofacitinib treatment., while IMT increased further despite treatment. Increased arginine and methionine might contribute to the anti-inflammatory effects of tofacitinib. Increased arginine availability with no changing ADMA may protect FMD and PWV from deterioration. The increase of IMT in the anti-inflammatory environment cannot be explained by arginine or methionine metabolism in this study.

## Introduction

**FIGURE 1:**
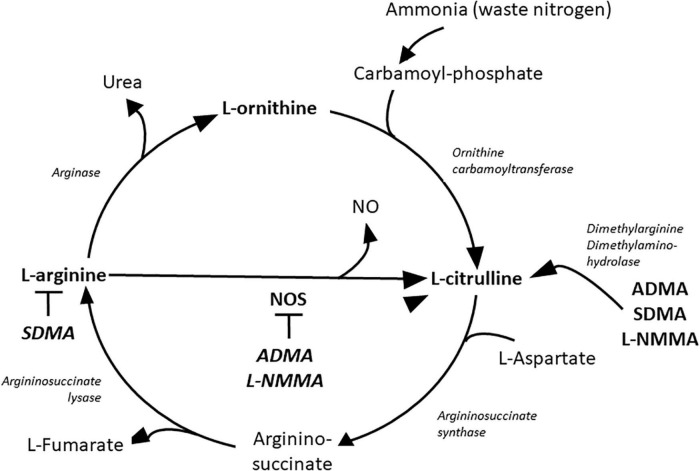
Metabolites of the urea cycle. ADMA, asymmetric dimethylarginine; L-NMMA, L-N-monomethyl-arginine; NO, nitric oxide; NOS, nitric oxide synthase; SDMA, symmetric dimethylarginine.

Rheumatoid arthritis (RA) is an autoimmune inflammatory rheumatic and musculoskeletal disease (RMD), which causes progressive deformation of different joints (1). Protein citrullination in the RA synovium is the first connection of the disease to arginine metabolism (2–5). At the joint level, there are known interactions between fibroblast-like synoviocytes (FLS), macrophages and helper T (T_*H*_)-, B- and plasma cells (6). In this environment, pro-inflammatory cytokines including tumor necrosis factor α (TNF-α), interleukin 1β (IL-1β) and IL-6 are abundantly produced (6, 7). This pro-inflammatory milieu promotes monocytes to differentiate to M1 type macrophages (6, 8). These macrophages express inducible nitric oxide synthase (iNOS) that stimulates the production of pro-inflammatory cytokines, NO and ONOO^–^ radicals at cytotoxic levels (9, 10). High expression of iNOS convert arginine into NO and citrulline (Figure 1) (10, 11). Synovial tissue damage initiates the differentiation of monocytes into M2 type macrophages (8), which cells produce large amount of arginase 1 and convert arginine to ornithine and urea (Figure 1) (10, 12, 13). Ornithine is converted to polyamines and proline promoting cell division and growth as well as collagen synthesis (11, 13). M2 macrophages produce anti-inflammatory cytokines and are involved in wound healing and tissue regeneration at the site of inflammation (8). The chronic inflammatory process is changing in time depending on which macrophages and cytokines are present and how the fibroblast-like synoviocytes (FLS) are activated (6–8). Pro-inflammatory cytokines can induce high amount of NO production with decreased arginase activation in human FLS (14–16). At the same time synovial M2 type macrophages show no increase in NO production but significant increase in arginase activity in the presence of pro-inflammatory cytokines (10, 12). The shift in the balance between iNOS and arginase 1 in the inflammatory process might shift the arginine metabolism either to citrulline or ornithine over-production, respectively (Figure 1) (10, 12).

Rheumatoid arthritis (RA), similarly to diabetes, obesity and hypertension, is associated with chronic systemic inflammation leading to endothelial dysfunction, accelerated inflammatory atherosclerosis manifesting in high risk of cardiovascular diseases (CVD) (17–20). In addition to coronary artery disease, endothelial dysfunction may cause myocardial hypertrophy and interstitial fibrosis manifesting in diastolic dysfunction and heart failure with preserved ejection fraction (EF) as also observed in diabetes, obesity and hypertension (21). The unique characteristic of CVD in RA is that there is higher risk for CVD at lower levels of cholesterol but high CRP levels. This lipid paradox may represent a special form of endothelial dysfunction where pro-inflammatory cytokines maintain the process of atherosclerosis (22, 23). Arginine is methylated during post-translational modification of different kind of proteins especially in the nucleus by protein methyl transferase enzymes (PRMT) (11). Three types of modifications are present on the proteins: N-monomethyl-L-arginine (L-NMMA), asymmetric (ADMA) and symmetric dimethyl arginine (SDMA) (11, 24). During protein turnover in the proteolysis phase the free L-NMMA and ADMA are metabolized to citrulline and dimethylamine by the enzyme dimethylarginine dimethylaminohydrolase (DDAH) (Figure 1) (11). Free SDMA is eliminated by the kidneys (25). L-NMMA and ADMA are competitive inhibitors of arginine at the NOS molecules causing decreased NO production and NOS uncoupling which turns NOS to produce superoxide anion instead of NO (11). ADMA and SDMA production has been associated with systemic inflammatory states (26–28). In arthritides, serum ADMA level is higher than normal and is associated with endothelial dysfunction (28–30). ADMA may cause endothelial dysfunction by disruption of the physiological endothelial NO production (11, 28–30). Physiological NO production inhibits platelet aggregation, tissue factor expression, expression of adhesion molecules, as well as smooth muscle cell (SMC) contraction, proliferation and migration (11). These physiological roles keep the endothelial layer intact and inhibit atherosclerosis or thrombus formation (11).

Non-invasive, ultrasound-based methods are able to evaluate endothelial function, such as flow mediated dilation of brachial artery (FMD) (19, 31). Endothelial dysfunction may enhance media thickening via SMC hypertrophy and proliferation, as well as media fibrosis leading to increasing arterial stiffness. This can be evaluated by aortic pulse wave velocity (PWV) changes (31). Similarly, overt atherosclerosis is reflected by increasing carotid intima media thickness (IMT) and the development of carotid plaques, which can be detected by B-mode ultrasound (19, 31).

**FIGURE 2:**
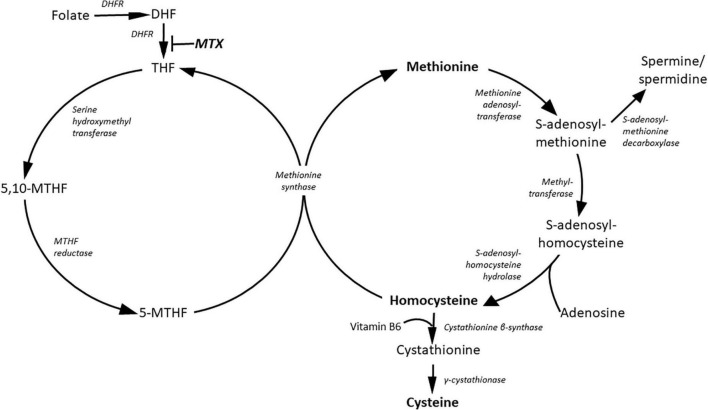
Metabolites of the methionine and folate cycles. DHF, dihydrofolate; DHFR, dihydrofolate reductase; MTHF, methylene-tetradhydrofolate; MTX, methotrexate; THF, tetrahydrofolate.

Methionine is the source of methylation by PMRT enzymes (Figure 2) (32). Homocysteine level is suppressed by cysteine synthesis, which enhance glutathione (GSH) synthesis and protection against oxidative stress (33). Increased methionine intake was associated with increased plasma concentration of homocysteine and reduced FMD in healthy human subjects (34, 35). The mechanism of inhibition of endothelial function by methionine load is not clear. Although the role of elevated ADMA level or oxidative stress were hypothesized they were not supported uniformly by various studies (34–36).

Methionine load with increasing plasma homocysteine concentration did not alter PWV although it changes aortic distensibility in healthy individuals (37). Population based interventional study using vitamin B12 and folic acid in hyperhomocysteinemic patients did not show any effect of decreasing homocysteine level on PWV or IMT (38).

Targeted therapies including biologic and targeted synthetic disease-modifying drugs dampen systemic inflammation, RA disease activity and they may also have beneficial effects on CV outcomes (17, 39, 40). As of today, four Janus kinase (JAK) inhibitors, tofacitinib, baricitinib, upadacitinib and filgotinib have been approved for the treatment of RA (41). JAK inhibition has been associated with elevation of lipids, possibly due to the lipid paradox described above (17, 42, 43). We have recently reported that tofacitinib dampened aortic wall inflammation by PET/CT (44). There has been only one study assessing IMT in tofacitinib-treated patients (45).

To our best knowledge there have been no other studies on the effects of JAK inhibitors on vascular pathophysiology measured by FMD, PWV and IMT in parallel with arginine and methionine metabolism. No previous studies evaluated arginine metabolism as an indicator of change in inflammation or endothelial dysfunction.

We conducted a prospective one-year study in order to assess the effects of tofacitinib on inflammation and functional status as well as FMD, PWV and IMT in RA patients. We propose that one-year tofacitinib treatment will, as a JAK-STAT inhibitor decrease pro-inflammatory cytokine levels, decrease inflammation and improve functional status with signals from methionine and arginine metabolism such as citrulline and ornithine levels. Improving inflammation may improve vascular status parallel with indicators of endothelial dysfunction in arginine metabolism like L-NMMA or ADMA levels.

## Patients and methods

### Patients and study design

Thirty patients with active RA were recruited for this tofacitinib interventional study. Patient characteristics are presented in Table 1. Inclusion criteria included definitive diagnosis of RA according to the 2010 European League Against Rheumatism (EULAR)/American College of Rheumatology (ACR) classification criteria for RA (1); moderate-high disease activity (DAS28 > 3.2) at baseline and clinical indication of targeted therapy. Patients were either naïve to any targeted therapies (*n* = 16) or initiated tofacitinib after stopping a biologic followed by an appropriate washout period (*n* = 14). Exclusion criteria included inflammatory diseases other than RA, acute/recent infection, standard contraindications to JAK inhibition, uncontrolled CV disease or hypertension, chronic renal or liver failure and malignancy within 10 years.

**TABLE 1:** Patient characteristics.

	Tofacitinib 5 mg bid	Tofacitinib 10 mg bid	Total
Number of recruited patients (n)	15	15	30
Female:male ratio	14:1	13:2	27:3
Age (mean ± SD) (range), years	52.3 ± 11.4 (27–69)	53.3 ± 8.8 (34–69)	52.8 ± 10.0 (27–69)
BMI (kg/m^2^)	29.5 ± 5.6 (22.0–40.2)	30.6 ± 8.6 (20.8–51.4)	30.0 ± 7.1 (20.8–51.4)
Positive CV history (n)	3	3	6
Positive history of hypertension (n)	6	8	14
Positive history of diabetes mellitus (n)	1	1	2
Smoking (current) (n)	4	3	7
Disease duration (mean ± SD) (range), years	6.3 ± 4.7 (1–15)	7.1 ± 4.9 (2–21)	7.7 ± 5.0 (1–21)
RF positivity, n (%)	12 (80)	12 (80)	24 (80)
Anti-CCP positivity, n (%)	13 (87)	11 (73)	24 (80)
DAS28 (baseline) (mean ± SD)	4.80 ± 0.69	5.29 ± 0.79	5.05 ± 0.77

BMI, body mass index; CCP, anti-cyclic citrullinated peptide; DAS28, 28-joint disease activity score; RF, rheumatoid factor; SD, standard deviation.

The 30 enrolled patients were randomly assigned in a 1:1 ratio to either 5mg or 10mg tofacitinib twice daily (bid) treatment arms. All patients received tofacitinib in combination with either methotrexate (MTX) (*n* = 23) or leflunomide (*n* = 7). MTX and leflunomide had been taken in stable dose at least one year prior to the present study. No dose changes of these DMARDs were allowed throughout the course of the study. Although most patients may have received corticosteroids prior to the study, none of the patients had been on corticosteroids for at least 3 months prior to and during the study.

Clinical assessments were performed at baseline, and after 6 and 12 months of therapy. Four patients (2 on each arm) completed the 6-month follow-up but did not complete the one-year treatment. Twenty-six patients completed the one-year treatment period and were included in the data analysis.

The study was approved by the Hungarian Scientific Research Council Ethical Committee (approval No. 56953-0/2015-EKL). Written informed consent was obtained from each patient and assessments were carried out according to the Declaration of Helsinki and its amendments.

### Clinical assessment

First, a detailed medical history was taken. We inquired about history of CVD, as well as current smoking, experience of chest pain resembling angina pectoris, hypertension and diabetes mellitus during the last 2 years prior to the start of this study by a questionnaire (Table 1). Further clinical assessments including physical examination were performed at baseline, and after 3, 6 and 12 months of tofacitinib therapy.

### Laboratory measurements and assessment of disease activity

Blood samples were drawn from fasting patients in the morning into ethylene-diamine-tetraacetate (EDTA)-treated tubes and were immediately processed, aliquoted and stored at –70°C until use. Blood samples were taken at baseline, as well as after 6 and 12 months of tofacitinib treatment.

Serum high sensitivity C reactive protein (hsCRP; normal: ≤5 mg/l) and IgM rheumatoid factor (RF; normal: ≤50 IU/ml) were measured by quantitative nephelometry (Cobas Mira Plus, Roche Diagnostics, Basel, Switzerland), using CRP and RF reagents (both Dialab Ltd, Budapest, Hungary). ACPA (CCP) autoantibodies were detected in serum samples using a second generation Immunoscan-RA CCP2 ELISA test (Euro Diagnostica, Malmö, Sweden; normal: ≤25 IU/ml). The assay was performed according to the manufacturer’s instructions.

Disease activity of RA was calculated as DAS28-CRP (3 variables) (46).

### Assessment of vascular physiology by ultrasound

Brachial artery FMD was assessed as described before (47). In brief, ultrasound examination was performed on the right arm using 10 MHz linear array transducer (ultrasound system: HP Sonos 5500, Hewlett Packard, Palo Alto, CA, USA) by a single trained sonographer after 30 min resting in a temperature-controlled room (basal value for FMD). A B-mode longitudinal section was obtained of the brachial artery above the antecubital fossa. In order to assess FMD, reactive hyperemia was induced by release of a pneumatic cuff around the forearm inflated to suprasystolic pressure for 4.5 min. After deflation the maximal flow velocity and the arterial diameter was continuously recorded for 90 s. Flow velocities, the baseline diameter, as well as FMD were ECG gated and detected offline. FMD values were expressed as% change from baseline (resting) value.

The IMT measurements were carried out as described before (47). Briefly, a duplex ultrasound system (HP Sonos 5500, 10 MHz linear array transducer) was used to assess the common carotid arteries by a single observer. Longitudinal high-resolution B-mode ultrasound scan were employed over both right and left common carotid arteries and were R-synchronized and recorded. The offline measurements were performed 1 cm proximal to the carotid bulb in the far wall. IMT was defined as the distance between the first and second echogenic lines from the lumen taking the average of 10 measurements on both sides. IMT values were expressed in mm.

With respect to arterial stiffness, PWV was calculated automatically by a TensioClinic arteriograph system (Tensiomed Ltd, Budapest, Hungary) as the quotient of the distance between the jugular fossa and symphysis as described before (47). If an artery is elastic, PWV is low. With decreased arterial elasticity, PWV rises. The arteriograph assesses this parameter from the oscillometric data obtained from the 35 mmHg suprasystolic pressure of the brachial artery. In order to obtain reproducible results, the patient had to rest in a supine position for at least 10 min before the assessment in a quiet room. PWV is expressed in m/s. Reproducibility of the three techniques expressed in intraclass correlation is included in the Statistical analysis section.

### Assessment of metabolites in the urea and methionine cycles

L-arginine, L-citrulline, L-NMMA, ADMA, SDMA, cysteine, homocysteine, methionine and ornithine were quantified using a validated HPLC-MS/MS assay (48, 49). In brief, 50 μl of internal standard containing solution (50 μM d7-ADMA, d7-arginine, d4-cysteine, d6-citrulline, d8-homocystine, d3-methionine and d6-ornithine, all in HPLC water) and 40 μl of 500 mM DTT solution were added to 100 μl serum. For protein precipitation, 400 μl of acetonitrile containing 0.1% trifluoric acid was added to the sample. The sample was vortexed for 5 min, centrifuged for 20 min at 16,000 *g*, and transferred into a HPLC vial. Ten μl of the supernatant was injected onto a 4.6 × 12.5-mm guard column (Eclipse XDB-C8, 5 μm, Agilent Technologies, Palo Alto, CA, USA) in line with a 3.0 × 150-mm analytical column (RP-Amide, 3.5 μm, Supelco, St. Louis, MO, USA). The API5000 mass spectrometer (AB Sciex, Concord, ON, Canada) was run in the positive electrospray ionization mode (ESI) using multiple reaction monitoring (MRM) (48, 49).

### Measurement of human inducible nitric oxide synthase

Inducible nitric oxide synthase (iNOS) was measured in human plasma by sandwich-enzyme-linked immunosorbent assay (ELISA) according to the manufacturer’s instructions (Novus Biologicals, CO, USA). The micro ELISA plate provided in the kit had been pre-coated with an antibody specific to human iNOS. Fifty micro liter of standard (serially diluted from 1000 to 15.6 pg/ml) or test sample was added to the plate wells and incubated for 2 h. This was followed by addition of 100 μl biotinylated detection antibody specific for human iNOS for 1 h and 100 μl of avidin-horseradish peroxidase (HRP) conjugate for 0.5 h. The substrate solution (90 μl) was added to each well for 20 min. The enzyme-substrate reaction was terminated by the addition of stop solution. Optical density (OD) was measured at a wavelength of 450 nm, Concentration of iNOS in the samples was calculated by comparing the OD of the samples to the standard curve. All incubations were done on a shaker at room temperature and plates were washed with wash buffer between incubation periods.

### Statistical analysis

Statistical analysis was performed using SPSS version 22.0 (IBM, Armonk, NY, USA) software. Data are expressed as the mean ± SD for continuous variables and percentages for categorical variables. The distribution of continuous variables was evaluated by Kolmogorov-Smirnov test. Continuous variables were evaluated by paired two-tailed *t*-test and Wilcoxon test. Nominal variables were compared between groups using the chi-squared or Fisher’s exact test, as appropriate. Correlations were determined by Pearson’s analysis. Univariate and multivariate regression analysis using the stepwise method were applied to investigate independent associations between metabolites of the urea or methionine cycle (dependent variables) and clinical (disease activity), laboratory (CRP, ESR) or vascular physiology parameters (independent variables). The β standardized linear coefficients showing linear correlations between two parameters were determined. The B (+95% CI) regression coefficient indicated independent associations between dependent and independent variables during changes. General linear model (GLM) repeated measures analysis of variance (RM-ANOVA) was performed in order to determine the additional effects of multiple parameters including therapy on 6- or 12-month changes of metabolite levels. In this analysis, partial η^2^ is given as indicator of effect size, with values of 0.01 suggesting small, 0.06 medium and 0.14 large effects. The power was estimated using the G*-Power 3 software (50). *P* < 0.05 were considered significant.

The reliability of the vascular ultrasound measurements was tested by inter-item correlation and intraclass correlation (ICC). With respect to the FMD, IMT and PWV tests, ICC = 0.470; F-test value: 1.887; *p* = 0.001. The power was estimated using the G*-Power software (50). *P* < 0.05 were considered significant.

## Results

### Characteristics of patients

Patient characteristics are seen in Table 1. Altogether 6 patients (3-3 on each arm) had a positive CV history. All these patients had coronary artery disease, which was properly treated and stable. A total of 14 patients had hypertension, 2 had diabetes mellitus and 7 had been current smokers at the time of inclusion. Hypertension and diabetes mellitus were also well-controlled in those patients (Table 1).

### Clinical response to tofacitinib therapy

Eventually a total of 4 patients, 2-2 each treatment arms, dropped out after 6 months of treatment but before the end of the study. Out of the 4 patients, 2 had inefficacy, one had significantly elevated transaminases and one moved abroad. Thus, 13-13 patients on each arm completed the study and were eligible for further data analysis (Table 1).

Tofacitinib treatment significantly decreased DAS28 after 6 months (3.31 ± 0.91; *p* < 0.001) and 12 months of treatment (3.32 ± 1.12; *p* < 0.001) compared to baseline (5.05 ± 0.77). CRP decreased from 14.8 ± 14.9 mg/l at baseline to 5.3 ± 5.3 mg/l after 6 months (*p* < 0.001) and 7.4 ± 7.7 mg/l after 12 months (*p* = 0.001). Similar observations were made in the 5 mg bid and 10 mg bid subsets (data not shown).

### Effects of tofacitinib on vascular pathophysiology

**FIGURE 3:**
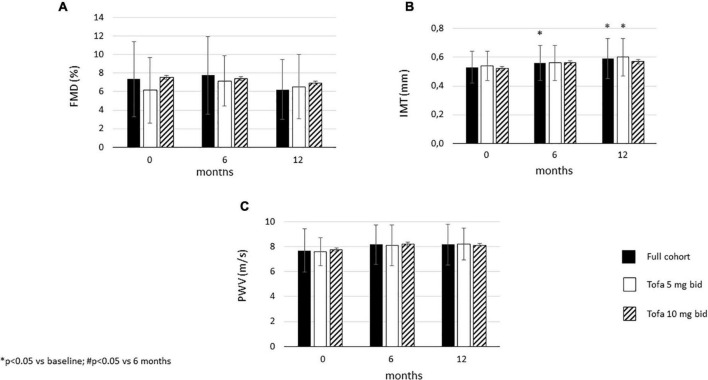
Effects of one-year tofacitinib therapy on **(A)** flow-mediated vasodilation (FMD). **(B)** carotid intima-media thickness (IMT) and **(C)** arterial pulse-wave velocity (PWV). In the full cohort and in the 5 mg bid group there was a progression in IMT after 12 months. In contrast, 10 mg bid tofacitinib was able to halt IMT progression. Both doses of tofacitinib slowed down FMD and PWV progression.

Carotid IMT significantly increased after 6 months (0.56 ± 0.12 mm; *p* = 0.05) and 12 months (0.59 ± 0.14 mm; *p* = 0.002) compared to baseline (0.53 ± 0.11 mm) in the full cohort. In the 5mg bid subset, there was no difference in IMT between 6 months and baseline. However, after 12 months, IMT significantly increased vs. baseline (*p* = 0.007). In the 10 mg bid subset, there were no significant differences in IMT after 6 or 12 months compared to baseline (Figure 3). In the total cohort, as well as in the 5 mg bid and 10 mg bid subsets, neither FMD nor PWV showed any significant changes over time (Figure 3).

### Effects of tofacitinib therapy on l-arginine metabolism

**FIGURE 4:**
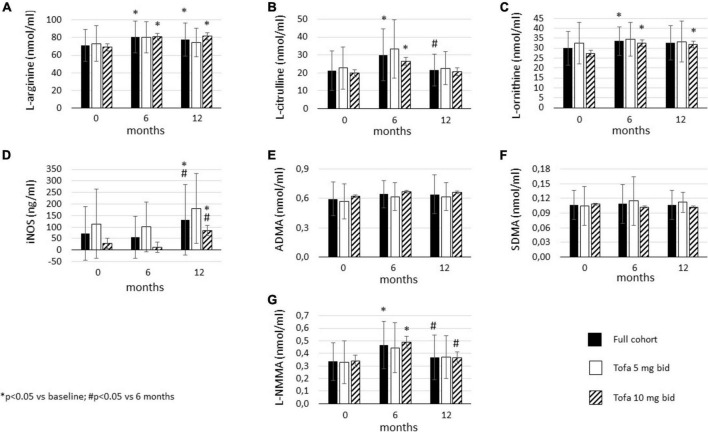
Effects of one-year tofacitinib therapy on metabolites in the urea cycle. **(A)** L-arginine levels increased in the full cohort and in the 10 mg bid subset after 6 and 12 months. **(B)** L-citrulline production transiently increased in the full cohort and the 10 mg bid subset after 6 months. **(C)** L-ornithine levels increased overtime in the full cohort and in the 10 mg bid subset after 6 and 12 months. **(D)** iNOS release increased in the full cohort and in the 10 mg bid subset after 12 months. **(E)** ADMA and **(F)** SDMA levels did not change overtime. **(G)** L-NMMA production transiently increased in the full cohort and the 10 mg bid subset after 6 months.

In the full cohort, tofacitinib significantly increased L-arginine levels by 6 (*p* = 0.004) and 12 months (*p* = 0.043). Similar pattern was observed in the 10 mg bid subset (*p* = 0.004 and *p* = 0.013, respectively) but not in the 5 mg bid subset (Figure 4A).

L-citrulline levels showed a transient increase in the full cohort. L-citrulline increased after 6 months vs. baseline (*p* = 0.006) but significantly decreased after 12 months compared to 6 months (*p* = 0.023). Tofacitinib 10 mg bid also transiently increased L-citrulline after 6 months (*p* = 0.018). No change was observed in the 5 mg bid subset (Figure 4B).

In the full cohort, L-ornithine also increased after 6 months vs. baseline (*p* = 0.025). Then, L-ornithine levels showed a tendency to remain higher after 12 months (*p* = 0.119). There was no significant difference between L-ornithine levels after 6 and 12 months. In the 10 mg bid subset, L-ornithine levels were significantly higher after both 6 (*p* = 0.018) and 12 months (*p* = 0.020) vs. baseline. Again, L-ornithine production did not change over time in the 5 mg bid subset (Figure 4C).

We also determined L-arginine/L-citrulline and L-arginine/L-ornithine ratios at 6 and 12 months. These ratios reflect the above described balances between pro- and anti-inflammatory mechanisms that may, at least in part, lead to clinical improvement upon tofacitinib treatment. Both L-arginine/L-citrulline (*p* = 0.007) and L-arginine/L-ornithine ratio (*p* = 0.035) significantly but transiently decreased after 6 months. Both ratios returned to the baseline level after 12 months (data not shown).

Inducible nitric oxide synthase (iNOS) levels after 12 months were significantly higher compared to both baseline (*p* = 0.045) and 6 months (*p* = 0.020) in the full cohort. There was no difference between 6 months and baseline. Similar pattern was observed in the 10 mg bid subset (*p* = 0.047 and *p* = 0.043, respectively), but not in the 5 mg bid subset (Figure 4D).

Tofacitinib treatment did not change ADMA (Figure 4E) or SDMA levels (Figure 4F) over time. No changes were seen in the full cohort, 5 mg or 10 mg bid subsets.

Finally, L-NMMA levels also showed a transient increase upon tofacitinib therapy. In the full cohort, L-NMMA was significantly increased after 6 months (*p* = 0.008), but then levels dropped after 12 months compared to 6 months (*p* = 0.048). Similar pattern was observed in the 10 mg bid subset (*p* = 0.044 and *p* = 0.047, respectively). There was no change in L-NMMA in the 5 mg bid subset (Figure 4G).

### Effect of tofacitinib on metabolites in the methionine cycle

**FIGURE 5:**
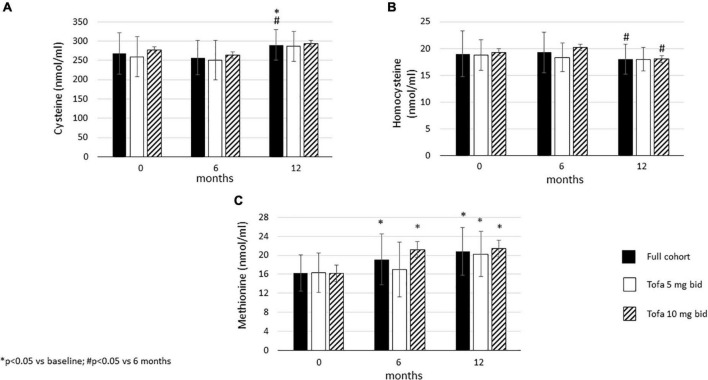
Effects of one-year tofacitinib therapy on metabolites in the methionine cycle. **(A)** Cysteine levels increased overtime in the full cohort after 12 months. **(B)** Homocysteine production decreased in the full cohort and the 10 mg bid subset after 12 months. **(C)** Methionine levels increased overtime in the full cohort and in the 10 mg bid subset after 6 and 12 months and also in the 5 mg bid subset after 12 months.

In the full cohort, cysteine showed a “late” increase after 12 months compared to baseline (*p* = 0.028) and 6 months (*p* = 0.005). There was no difference between baseline and 6 months. This increase was not observed in the 5 mg bid or 10 mg bid subset (Figure 5A).

Homocysteine levels did not change between 6 months and baseline, however, later significant decreases were observed between 6 and 12 months in the full cohort (*p* = 0.047), as well as in the 10 mg bid subset (*p* = 0.049). No changes in homocysteine levels were seen in the 5 mg bid subset (Figure 5B).

Finally, methionine levels increased after 6 months versus baseline in the full cohort (*p* = 0.005) and in the 10 mg bid subset (*p* < 0.001). In addition, methionine levels increased after 12 months compared to baseline in the full cohort (p < 0.001), as well as in the 5 mg bid (*p* = 0.002) and the 10 mg bid arms (*p* < 0.001) (Figure 5C).

### Correlations between levels of metabolites with each other and with other parameters

Table 2 shows uni- and multivariate regression analysis of metabolites as dependent variables. In general, the univariate analysis suggests that baseline and 12-month L-arginine, L-citrulline, L-NMMA, ADMA, homocysteine and methionine levels variably correlate with disease activity/inflammation (DAS28, CRP, ESR) or seropositivity (RF, CCP). The correlations with DAS28 are positive, while those with CRP, ESR and CCP are negative. RF positively correlated with L-arginine and L-citrulline, but negatively with ADMA, L-NMMA and homocysteine. Baseline disease duration may also determine L-NMMA after 12 months.

**TABLE 2:** Univariable and multivariable analysis of determinants of metabolites as dependent variables.

Dependent variable	Independent variable	Univariable regression analysis				Multivariable regression analysis			
		β	p	B	95% CI	β	p	B	95% CI
*L-arginine-0*	*CRP-0*	–0.386	0.041	–0.441	–0.886 – 0.003				
*L-arginine-12*	*CRP-0*	–0.386	0.041	–0.441	–0.886 – 0.003				
	*CRP-12*	–0.392	0.047	–0.888	–1.766 to –0.011	–0.522	0.004	–1.182	–1.943 to –0.421
	*RF-12*	0.413	0.036	0.044	0.003 – 0.084	0.539	0.003	0.057	0.021 – 0.093
*L-citrulline-0*	*CRP-0*	–0.421	0.032	–0.296	–0.564 to –0.027				
	*ESR-0*	–0.432	0.027	–0.226	–0.426 to –0.027	–0.432	0.027	–0.226	–0.426 to –0.027
*L-citrulline-12*	*RF-0*	0.585	0.002	0.024	0.010 – 0.038	0.585	0.002	0.024	0.010 – 0.038
	*RF-12*	0.441	0.024	0.022	0.003 – 0.042				
*L-ornithine-0*	–								
*L-ornithine-12*	*DAS28-12*	–0.430	0.028	–3.968	–7.477 to –0.458				
*iNOS-0*	–								
*iNOS-12*	–								
*ADMA-0*	*CRP-0*	–0.563	0.003	–0.006	–0.010 to –0.002	–0.563	0.003	–0.006	–0.010 to –0.002
	*ESR-0*	–0.496	0.010	–0.004	–0.007 to –0.001				
	*aCCP-0*	–0.456	0.019	0	0 – 0				
	*RF-0*	–0.467	0.016	0	–0.001 – 0				
*ADMA-12*	*DAS28-12*	0.465	0.017	0.099	0.020 – 0.178	0.544	<0.001	0.116	0.061 – 0.171
	*CRP-0*	–0.594	0.001	–0.007	–0.012 to –0.003	–0.659	<0.001	–0.008	–0.012 - -0.005
	*CRP-12*	–0.421	0.032	–0.010	–0.020 to –0.001				
	*ESR-0*	–0.379	0.046	–0.004	–0.007 – 0				
	*aCCP-0*	–0.488	0.011	0	0 – 0				
	*aCCP-12*	–0.508	0.008	0	0 – 0				
*SDMA-0*	–								
*SDMA-12*	–								
*L-NMAA-0*	*ESR-0*	–0.406	0.040	–0.003	–0.006 – 0				
	*RF-0*	–0.465	0.017	0	–0.001 – 0				
*L-NMAA-12*	*disease duration*	0.462	0.017	0.016	0.003 – 0.029	0.383	0.028	0.014	0.002 – 0.025
	*CRP-12*	–0.517	0.007	–0.011	–0.019 to –0.003	–0.449	0.011	–0.010	–0.017 - -0.002
*Cysteine-0*	–								
*Cysteine-12*	–								
*Homocysteine-0*	*ESR-0*	–0.517	0.007	–0.105	–0.178 to –0.032				
*Homocysteine-12*	*CRP-0*	–0.428	0.029	–0.074	–0.140 to –0.008				
	*aCCP-0*	–0.386	0.042	–0.001	–0.002 – 0				
	*aCCP-12*	–0.439	0.025	–0.001	–0.002 – 0				
	*RF-12*	–0.449	0.021	–0.003	–0.013 to –0.001	–0.449	0.021	–0.003	–0.013 - -0.001
*Methionine-0*	*CRP-0*	–0.412	0.037	–0.105	–0.193 to –0.007				
*Methionine-12*	*DAS28-0*	–0.417	0.034	–2.739	–5.252 to –0.225	–0.433	0.012	–2.844	–4.345 – -0.334
	*CRP-12*	–0.398	0.044	–0.241	–0.476 to –0.007				
	*PWV-12*	0.486	0.012	1.472	0.352 – 2.598	0.498	0.005	1.515	0.517 – 2.512

aCCP, anti-cyclic citrullinated peptide; ADMA, asymmetric dimethylarginine; CI, confidence interval; CRP, C-reactive protein; DAS28, 28-joint disease activity score; ESR, erythrocyte sedimentation rate; FMD, flow-mediated vasodilation; iNOS, inducible nitric oxide synthase; L-NMMA, L-N-monomethyl arginine; PWV, pulse-wave velocity; RF, rheumatoid factor; SDMA, symmetric dimethylarginine.

There were sporadic correlations between vascular parameters and arginine or methionine metabolites. FMD negatively correlated with arginine and methionine at baseline. IMT positively correlated with ADMA and L-NMMA at 6 months. PWV positively correlated with homocysteine at baseline and methionine at 12 months.

The multivariate analysis confirmed variable correlations of L-arginine, L-citrulline, ADMA, L-NMMA, homocysteine and methionine after one-year treatment with DAS28, CRP, ESR and RF, but not with anti-CCP. Interestingly, most correlations, except for two, were observed regarding 12-month levels of the urea and methionine cycle metabolites. With respect to vascular pathophysiology, only PWV and methionine after 12 months correlated with each other (Table 2).

Repeated measures analysis of variance (RM-ANOVA) was performed in order to determine the effects of tofacitinib treatment in combination with other parameters on changes in the levels of metabolites over time. Six-month change (increase) in L-citrulline levels was significantly determined by tofacitinib treatment together with either lower ESR or higher RF at baseline. After 6 months, L-citrulline decreased to the level of baseline. Moreover, 12-month changes in homocysteine levels correlated with treatment in combination with lower ESR at baseline (Table 3).

**TABLE 3:** Significant results of general linear model (GLM) repeated measures analysis of variance (RM-ANOVA) test determining the effects of treatment and other independent variables on 12-month changes in the levels of metabolites as dependent variables.

Dependent variable	Effect	F	p	Partial η^2^
*L-citrulline 0-6*	treatment * lower ESR-0 treatment * higher RF-0	8.855 22.578	0.007 <0.001	0.270 0.485
*Homocysteine 0-12*	treatment * lower ESR-0	5.273	0.031	0.180

ESR, erythrocyte sedimentation rate; RF, rheumatoid factor.

## Discussion

Rheumatoid arthritis (RA) is characterized by systemic inflammation and high risk for CVD. In the present study, we have followed endothelial function (FMD), vascular stiffness (PWV) and intima-media thickness (IMT) during one-year treatment with tofacitinib. We applied a metabolomic approach in order to evaluate the dose dependent effect of tofacitinib treatment on arginine and methionine metabolism in association with the level of inflammation and vascular pathology. The two amino acids are interacting with each other as methionine is the source of methylation of arginine on proteins and alters homocysteine and cysteine synthesis (51–53).

One-year tofacitinib treatment decreased CRP, ESR and DAS28 showing significant antiinflammatory effects and improving functional status.

Tofacitinib has significantly increased arginine and methionine levels during the one-year treatment. Inflammation with increased Il-6 level decreases muscle protein synthesis and increases amino-acid uptake of other organs decreasing the amino-acid level (54). As tofacitinib, among other cytokines, suppresses IL-6 production it might potentially reverse the effect described above, promoting an increase in plasma level of amino acids.

Citrulline and ornithine level may represent the balance between inflammatory and anti-inflammatory macrophages or other cell types. Citrulline and ornithine levels increased significantly at 6 months and returned to baseline in the whole group. The increasing citrulline may represent flaring inflammation with producing destructive NO production by M1 type macrophages or other inflammatory cells. The decrease of CRP, ESR and DAS28 at 6 months represented inhibition of inflammation, thus the increased NO production most likely belonged to endothelial cells. The increased ornithine level, however, may represent increased arginase activity in M2 type macrophages in parallel with the anti-inflammatory effect of tofacitinib. We do not have enough data to explain why citrulline and ornithine levels returned to baseline at 12 months and what is the clinical significance of the significantly higher level of ornithine in the 10mg BID tofacitinib group at 12 months. There has been only one cross-sectional study assessing metabolites of arginine in RA (27). In that study, decreased levels of plasma arginine and citrulline, elevated levels of L-ornithine and elevated arginase activity was found in RA patients compared to controls. In that patient group the CRP and ESR levels were almost normal so those patients represented marked anti-inflammatory macrophage activity with increased arginase level. In our patient group the CRP and ESR values were high even after tofacitinib treatment, which might explain we did not see decreasing arginine and major shift in the ornithine and citrulline ratio.

Inducible nitric oxide synthase (iNOS) level was significantly higher at 12 months in the whole ant the 10mg BID groups. In an *in vitro* observation of the effect of tofacitinib on human dendritic cells and macrophages, tofacitinib induced M1 macrophage phenotypes with iNOS expression and IL-12 and IL-23 production (55). However, it should be considered that tofacitinib was combined with methotrexate in our study. Methotrexate inhibits M1 and promotes M2 macrophage differentiation through adenosine receptors. At the same time it is JAK1/JAK2 inhibitor also (56). Our study suggests that the interaction between methotrexate and tofacitinib might shift to M2 macrophage activity with slight increase in arginase activity and ornithine level and inhibition of the cells with iNOS expression especially at higher dose of tofacitinib.

Similarly to arginine, methionine level significantly increased at 6 and 12 months. Methionine, as an essential amino acid, is highly involved in the methylation of different proteins and nucleic acid (32). Methylations could modulate T cell functions, impairing Th1/Th2 cytokines release, and decreasing T cell proliferation and activation. Furthermore, epigenetic regulations play a significant role in gene expression affecting immunocyte function and signaling pathways (32). Global DNA hypomethylation in RA synovial fibroblasts contributes to their intrinsic activation (57). In our study the increased methionine availability during tofacitinib treatment may provide at least appropriate methylation status mediating anti-inflammatory effects. However, homocysteine level did not change significantly from the baseline level with increasing methionine indicating the same level of methylation reactions. Following methionine and homocysteine levels are not conclusive to describe the change in inflammation during tofacitinib treatment. There was an increase in cysteine and a decrease in homocysteine synthesis at 12 months. Increased cysteine synthesis initiates more glutathione production, which enhance cellular protection against oxidative stress (58).

Tofacitinib therapy did not alter endothelial function and vascular stiffness measured by FMD and PWV, respectively. In most studies, impairment of FMD (19, 20, 31, 47, 59, 60) and increasing PWV (31, 47, 61–63) were observed in RA. Our study suggests that one year tofacitinib therapy was able to prevent further worsening of endothelial function and arterial stiffness, respectively. In contrast, carotid atherosclerosis (IMT) further deteriorated overtime despite tofacitinib therapy. In numerous studies, IMT was between 0.63 and 0.73 mm in RA compared to 0.54-0.62 mm in healthy controls (19, 20, 31, 64–66). In our present study, the mean baseline IMT was only 0.53 mm (range: 0.40–0.66 mm), which is similar to healthy subjects in other studies. Although, IMT progressed despite tofacitinib treatment, it does not mean that tofacitinib would promote atherosclerosis. One year of tofacitinib treatment may not be long enough to suppress atherosclerosis. Very recently, in the ORAL Surveillance study, tofacitinib treatment in comparison to anti-TNF therapy was associated with increase risk of major cardiovascular events (67). That study, on contrast to ours, included RA patients with at least one cardiovascular risk at baseline and the average age of that patient group was 10-year older, than ours. The latest integrated safety analysis did not find increased cardiovascular risk upon tofacitinib therapy (68).

One-year tofacitinib treatment decreased CRP, ESR and DAS28 showing significant antinflammatory effects but it did not show major effect on vascular function. There has been only one study where IMT did not significantly change during one year tofacitinib treatment (10mg/day) in a methotrexate resistant patient group. In this study cardio-ankle vascular index and augmentation index decreased significantly after one-year treatment (45). The notable difference between their study and ours, that the CRP level was twice higher in our study at 1 year. This might explain why only 20mg/day tofacitinib were effective in our study to inhibit significant increase in IMT. We did not find any other studies where tofacitinib or any other JAK inhibitors were studied in relation to FMD or PWV. Other investigators also did not find association between vascular function or morphology and ADMA or SDMA in RA (69, 70).

Arginine, citrulline, ornithine and global arginine bioavailability ratio (GABR) [arginine/(citrulline + ornithine)] were measured in patients with and without coronary artery disease (CAD). The prevalence of CAD and risk of death or major adverse cardiovascular events (MACE) were evaluated at 3 years with decreasing quartiles of arginine and GABR and increasing quartiles of citrulline and ornithine. The best predictor for CAD, MACE and death was GABR and >1.46 characterized the smallest risk. This study suggested that arginine availability is an important predictor in vascular pathology (71). In our study, the calculated GABR was 1.47 ± 0.41, 1.31 ± 0.36, 1.49 ± 0.38 at 0, 6, and 12 months, respectively. These numbers put our patient group in low-risk categories for CAD, death and MACE. The individual concentrations of arginine, citrulline and ornithine represented low risk categories also during tofacitinib treatment. This may explain that tofacitinib has not altered endothelial function (FMD) and vascular stiffness (PWV) during one-year treatment. At higher concentration tofacitinib inhibited carotid atherosclerosis maybe because that patient group had the highest arginine bioavailability. We could not find any correlation between the vascular parameters and arginine or methionine metabolism, most likely because the measured parameters were in the normal range. This study was not controlled, however, as a historical control, previous studies indicated that impaired FMD and increased IMT and PWV have been associated with RA (17, 18, 31, 39, 47, 72) and biologics including TNF blockers and others may, at least transiently, improve endothelial function, atherosclerosis and arterial stiffness or at least slow down the progression of these parameters [reviewed in Szekanecz et al. (39)].

Methylated arginine derivatives are enriched in the plasma after proteolysis (73–75) and the DDAH enzyme transforms ADMA and L-NMMA to L-citrulline (73). Increased circulating ADMA levels have been associated with RA and spondyloarthritides (28, 29). Similarly to increasing citrulline and ornithine, increasing ADMA level increased the risk for MACE in a patient groups of peripheral artery disease or in CAD (30, 76, 77). In patients with peripheral artery disease ADMA level < 0.68 μmol/l represented the lowest quartile for hazard ratio for MACE. In our study the ADMA level was between 0.6 and 0.64 μmol/l range representing a normal level and low risk for MACE. This ADMA level did not change significantly during the study period. The effects of targeted therapies on ADMA may be controversial (24, 78). TNF-α inhibits ADMA degradation by inhibition of DDAH and thus increases ADMA levels (78). Anti-TNF therapy decreased ADMA levels in a few (78) but, similarly to ours, not in most studies (79–81). The L-arginine/ADMA ratio is important for modulation of NOS activity and it is a risk factor for atherosclerosis (82, 83). TNF-α inhibitors may increase L-arginine/ADMA ratios at 3 and 12 months of treatment (84). We observed a similar pattern in our present study applying tofacitinib therapy. Although the L-arginine/ADMA ratio did not change significantly in our study, the higher L-arginine levels along with unchanged levels of ADMA might contribute to the inhibition of carotid wall thickening at the higher dose of tofacitinib therapy. We have recently reported that tofacitinib attenuated vascular inflammation as determined by PET/CT (44). Based on our study the increased arginine level, high arginine availability for NO synthesis and high arginine/ADMA ratio may explain the attenuation of vascular inflammation during tofacitinib treatment.

Although, methionine showed a negative correlation with FMD at baseline, increased methionine level at 6 and 12 months did not change FMD significantly. The effect of chronic elevation of methionine on FMD is different from the results of previous studies where methionine load increased homocysteine level and decreased FMD in healthy individuals and patients with various diseases (34–36). Elevated homocysteine and cysteine levels were associated with decreased FMD across menopausal stages in healthy women. In this study methionine showed a positive correlation with FMD (49). Homocysteine level is higher in RA compared to normal population (85). In our longitudinal study homocysteine level was higher than normal and there was no significant difference between baseline and 12-month values although the methionine level increased significantly. In conclusion, tofacitinib treatment increase methionine level without altering homocysteine level and endothelial function. There was a positive correlation between methionine and PWV at 12 month in our study. On the other hand, there was no significant change in PWV at 12 months compared to baseline values. In comparison with FMD, PWV is not changing with methionine load (37). However, multiple studies showed association between homocysteine and PWV in various diseases (86–88). One year tofacitinib treatment did not alter PWV significantly in the presence of high homocysteine and methionine level suggesting not progressing vascular stiffness in our patient population.

Arginine, citrulline, ADMA, L-NMMA, homocysteine and methionine variably correlated with DAS28, CRP and ESR. Interestingly, these metabolites showed inverse correlations with CRP, a marker of systemic inflammation suggesting that the levels of some of these metabolites increase in parallel with attenuation of CRP levels by JAK inhibition. Some reports also found low levels of arginine and citrulline in severe infectious and inflammatory states associated with elevated CRP release (89, 90).

Our study has certain advantages and limitations. There have been no similar prospective studies assessing metabolites of the urea and methionine cycle in association with disease activity, autoantibodies and vascular pathophysiology in RA patients undergoing any targeted treatments. Possible limitations may include the relatively low number of RA patients and the lack of control group due to the fact that this is a self-controlled prospective study.

In conclusion, we propose that in our RA cohort, tofacitinib treatment, in parallel with its anti-inflammatory action, normalized the balance of amino acid metabolism and protected vascular function at least for one year. Although we did not assess M1 and M2 macrophages our results suggested that tofacitinib might shift the arginine metabolism to anti-inflammatory direction. The higher methionine level might also contribute to anti-inflammatory immune modulation. Endothelial function and arterial stiffness have not changed in one-year tofacitinib treatment partly due to the high arginine availability and not increasing ADMA level. Increasing methionine level did not associated with higher homocysteine level and did not alter FMD and PWV. The mechanism of increasing IMT during tofacitinib treatment needs further investigation.

## Data availability statement

The raw data supporting the conclusions of this article will be made available by the authors, without undue reservation.

## Ethics statement

The studies involving human participants were reviewed and approved by Hungarian Scientific Research Council Ethical Committee (approval No. 56953-0/2015-EKL). The patients/participants provided their written informed consent to participate in this study.

## Author contributions

BS: study concept, patient recruitment and examination, and manuscript drafting. AH, EV, SSzam, ZP, KG, SSzán, and GS: patient recruitment and examination, and data collection. AP and MC: laboratory tests and data analysis. GK: vascular assessments and data analysis. UC and JK: metabolic assessments, data analysis, and interpretation. TS and ZS: senior PIs, supervisors, study conceptualization, and manuscript finalization. All authors contributed to the article and approved the submitted version.
